# Revascularization strategy for left main coronary artery disease comparing percutaneous coronary intervention versus coronary artery bypass grafting

**DOI:** 10.1038/s43856-025-01098-w

**Published:** 2025-09-29

**Authors:** Ryaan EL-Andari, Jimmy Kang, Nicholas Fialka, Yongzhe Hong, Finlay A. McAlister, Jeevan Nagendran, Jayan Nagendran

**Affiliations:** 1 Division of Cardiac Surgery, Department of Surgery, University of Alberta, Edmonton, AB Canada; 2 Faculty of Medicine and Dentistry, University of Alberta, Edmonton, AB Canada

**Keywords:** Cardiology, Health care

## Abstract

**Background:**

Coronary artery bypass grafting(CABG) has long been the preferred treatment for left main coronary artery disease(LMCAD), although percutaneous coronary intervention(PCI) has been increasingly utilized. Despite numerous investigations seeking to identify the optimal revascularization strategy for LMCAD, limitations in sample size or follow-up duration have hindered definitive conclusions. Herein, we compare the long-term outcomes up to 14 years after CABG or PCI for patients with LMCAD.

**Methods:**

Data was retrospectively collected from a provincial database. The inclusion criteria is patients ≥18 years old, with LMCAD, and revascularization with CABG or PCI. The primary outcome is all-cause mortality. Secondary outcomes are any rehospitalization, myocardial infarction (MI), stroke, or repeat revascularization. Outcomes are adjusted for age, sex, and clinical comorbidities. The average age of the patients was 67 ± 9 years for the CABG patients and 71 ± 11 years for the PCI patients. 84.7% of the CABG patients and 71.5% of the PCI patients were male.

**Results:**

5580 patients are identified with LMCAD between 2009 and 2018. 1706 patients (1180 CABG; 526 PCI) are included in the final analysis and followed until March 31, 2023. Rates of mortality at longest follow-up of 14 years are 40.0% for CABG and 58.4% for PCI (adjusted hazard ratio(aHR) 0.58, 95% confidence interval(CI) 0.48–0.70, *p* < 0.001). Rates of MI (10.7% vs 22.3%, aHR 0.40, 95% CI 0.29–0.55, *p* < 0.001) and repeat revascularization (5.4%vs16.3%, aHR 0.25, 95% CI 0.18–0.36, *p* < 0.001) favor CABG over PCI.

**Conclusions:**

Patients with LMCAD undergoing CABG experience significant benefit over PCI in terms of long-term mortality, MI, and required repeat revascularization. These finding suggest CABG should remain the preferred revascularization strategy for patients with LMCAD and acceptable surgical risk. Future studies should explore evolving PCI techniques and their impact on long-term outcomes.

## Plain Language Summary

Coronary artery bypass grafting (CABG) or bypass surgery involves using vessels to bypass blockages in heart arteries. CABG is the preferred treatment for left main coronary artery disease (LMCAD), disease of one of the major arteries that supplies blood to a large area of the heart, although percutaneous coronary intervention (PCI) which involves artery stenting has been increasingly utilized. Previous studies comparing the two treatments in LMCAD have been limited in sample size or follow-up duration. This study compared the long-term outcomes up to 14 years after CABG or PCI for patients with LMCAD. 1706 patients (1180 CABG; 526 PCI) were compared. At longest follow-up, rates of death, heart attack, and repeat procedures were significantly lower for CABG compared to PCI. These finding suggest CABG should remain the preferred revascularization strategy for patients with LMCAD and acceptable surgical risk.

## Abstract (web summary)

EL-Andari et al. compare the outcomes of 1706 patients with left main coronary artery disease undergoing percutaneous coronary intervention (PCI) or Coronary artery bypass grafting (CABG) with up to 14-year follow-up. Findings show that patients who underwent CABG have reduced mortality, myocardial infarction and require repeat revascularization.

## Introduction

Coronary artery disease (CAD) is widely prevalent and increasing in frequency. It is estimated that 126 million people worldwide are afflicted with CAD and the prevalence of CAD in the United States is 7.2% for those over the age of 20^1,2^. Treatment options for severe CAD include medical therapy, percutaneous coronary intervention (PCI), and coronary artery bypass grafting (CABG). Numerous factors impact patient prognosis and response to therapy including preoperative comorbidities, and frailty, among others. Coronary anatomy is a significant consideration in identifying the optimal management of CAD. Patients with more complex CAD tend to benefit from CABG while patients with less complex CAD tend to benefit from PCI^3^. Features such vessel occlusion, multivessel CAD, disease at bifurcations, and significant calcium burden are some of the factors that increase disease complexity^3^.

One anatomical feature where controversy persists regarding the optimal revascularization strategy is patients with LMCAD. LMCAD is distinct from other anatomical features due to a lower threshold for intervention, its impact on a large area of myocardium, and its involvement of the left main bifurcation which increases technical complexity in PCI^4^. The SYNTAX Trial demonstrated benefit for patients with more complex CAD that received CABG compared to PCI, with this benefit being attenuated with less complex CAD^3,5^. The guidelines have in part reflected this finding with CABG receiving a class 1 indication encouraging use in high complexity CAD with LMCAD given the lower rates of mortality and repeat coronary events identified with CABG compared to PCI in the previous literature^6^. However, the benefits of CABG compared to PCI have not been consistent in patients with isolated LMCAD^1,7,8^. Previous investigations into this topic have had several limitations. Many of the comparisons between CABG and PCI have included small sample sizes, limited follow-up duration to less than 5 years, and with contrasting results such as non-inferiority identified for the primary outcome of the EXCEL Trial but inferior secondary outcomes for PCI^5,9–12^. With increasing utilization of PCI and limited follow-up available in the literature, controversy continues to exist with respect to the optimal management of this patient population. The addition of an adequately powered study with long-term follow-up is paramount in the identification of the optimal revascularization strategies for this patient population. As the optimal initial revascularization modality has the potential to impact long-term survival and recurrent coronary events, the current literature does not adequately inform these clinical decisions and guideline recommendations. Herein, we compare the long-term outcomes of patients with LMCAD undergoing PCI or CABG with longest follow-up of up to 14 years and identified reduced rates of mortality, myocardial infarction, and required repeat revascularization for patients that underwent CABG compared to PCI.

## Methods

### Data source

All preoperative and postoperative data for this retrospective cohort study were collected for this study from the discharge abstract databases and the APPROACH (Alberta Provincial Project for Outcome Assessment in Coronary Heart Disease) database. The date of death was collected from the Provincial Death Registry. The APPROACH database collects results of angiography, preoperative comorbidities, surgical or procedural details, and postoperative outcomes information from all patients in the province of Alberta undergoing coronary angiography. The data was obtained through application to the databases requesting specific data on this patient cohort. This study was approved by the local University of Alberta research ethics board under study ID Pro00125019 with a waiver of individual signed consent given the large number of patients and extended period of inclusion.

### Study cohort

The inclusion criteria for this study included Alberta residents who were 18 years or older and diagnosed with LMCAD with any number of diseased coronary arteries between January 1st, 2009 and December 31, 2018, and was followed by revascularization via PCI or CABG within 365 days. The average age of the patients was 67 ± 9 years for the CABG patients and 71 ± 11 years for the PCI patients. 84.7% of the CABG patients and 71.5% of the PCI patients were male. The exclusion criteria included patients who presented with ST-elevation MI (STEMI), a previous history of PCI or CABG, underwent concomitant valve surgery at the time of CABG, or were undergoing repeat revascularization up to the longest available follow-up. Patients with STEMI were excluded given their higher risk profile and propensity to be referred for PCI emergently. We also sought to directly compare revascularization strategies and so concomitant procedures or previous interventions were excluded in order to directly compare primary revascularization strategies.

All comorbidities were defined based on APPROACH forms and data. Significant lesions of the coronary arteries were defined as ≥50% stenosis within the APPROACH database during encoding, although >70% is often used as a threshold for treatment for vessels other than the left main coronary artery. The last date of follow-up was March 31, 2023. The index revascularization was defined as the CABG or PCI within 365 days of the angiogram. If a patient had multiple angiograms and were matched to the same revascularization, only the angiogram that was closest to the date of the revascularization would be considered as referred for the revascularization, otherwise would be deemed as receiving medical treatment alone. If an angiogram was linked to multiple revascularizations, only the first intervention would be treated as the index revascularization.

### Outcomes

The primary outcome was all-cause mortality during the study period. Secondary outcomes were rehospitalization for any cause, MI (ICD-10 codes I21, I22, I23 in the primary diagnosis of the readmission), stroke (ICD-10 codes I60, I61, I63, I64, and G45 in the primary diagnosis of the readmission), or repeat revascularization. Rates of the individual outcomes were captured in cases of admission where another condition was considered the primary cause of readmission, such as an MI resulting in heart failure, where both diagnoses would be captured regardless of the primary cause for readmission. Secondary outcomes were defined based on the admitting diagnosis for readmission at any time and at any acute care hospital in Alberta during the follow-up period.

### Statistics and reproducibility

Continuous variables and binary variables were reported as mean ± standard deviation (SD) and count (percent) respectively. The secondary aim of this study was to compare the rates of referral for PCI and CABG for these patients. Continuous variables were reported as mean ± SD and categorical variables as frequency (percent). Continuous variables were compared using t-test. Categorical variables were compared with Chi-square test or the Fisher exact test as appropriate.

Cox proportional hazards regression models and Fine & Gray model^13^ were implemented to estimate the hazard ratios of the difference in the fatal and non-fatal outcomes at the longest follow-up while adjusting for the covariates listed in baseline characteristics (Table 1). A Kaplan–Meier curve was plotted for all-cause mortality and cumulative incidence curves were also provided for all the non-fatal outcomes. Statistical analyses were executed using SAS 9.4 (SAS Institute, Cary NC). A *p* < 0.05 was deemed statistically significant. All statistical tests were two-sided.

**Table 1:** Baseline characteristics of patients with LMCAD that underwent CABG or PCI

Variables	CABG (*N* = 1180)	PCI (*N* = 526)	*P* value
Age, years	67 ± 9	71 ± 11	**6.94e-16**
Female	181 (15.3)	150 (28.5)	**2.07e-10**
Hypertension	690 (58.5)	306 (58.2)	0.908
Dyslipidemia	720 (61)	314 (59.7)	0.606
Heart failure	36 (3.1)	44 (8.4)	**1.63e-06**
Atrial fibrillation	3 (0.3)	5 (1)	0.052
Type I or II Diabetes	301 (25.5)	143 (27.2)	0.466
COPD	67 (5.7)	43 (8.2)	0.053
CEVD	29 (2.5)	22 (4.2)	0.053
CKD	6 (0.5)	11 (2.1)	**0.002**
PAD	38 (3.2)	24 (4.6)	0.171
Any Liver disease	0 (0)	1 (0.2)	0.134
Malignancy	4 (0.3)	12 (2.3)	**0.0001**
Current smoker	228 (19.3)	78 (14.8)	**0.026**
Former smoker	326 (27.6)	145 (27.6)	0.979
*Baseline Medications*			
ASA	787 (66.7)	348 (66.2)	0.829
Other antiplatelets	305 (25.8)	187 (35.6)	**4.39e-05**
Statin	626 (53.1)	290 (55.1)	0.426
ACEi/ARB	569 (48.2)	277 (52.7)	0.09
Beta-blocker	634 (53.7)	300 (57)	0.205
Anticoagulant	81 (6.9)	67 (12.7)	**6.89e-05**

Bold values indicate statistical significance.

*CABG* coronary artery bypass grafting, *PCI* percutaneous coronary intervention, *COPD* chronic obstructive pulmonary disease, *CEVD* cerebrovascular disease, *CKD* chronic kidney disease, *PAD* Peripheral arterial disease, *ASA* acetylsalicylic acid, *ACEi/ARB* Angiotensin-converting enzyme inhibitors/Angiotensin receptor blockers.

Propensity score matching methods were applied to control for the difference in the baseline covariates. The propensity score was estimated using a multivariable logistic regression model with revascularization type (PCI or CABG) as the dependent variable and the baseline characteristics as covariates including all the predictors in Supplementary Table 1. Greedy matching techniques without replacement and a caliper width equal to 0.1 of the standard deviation of the logit of the propensity score were applied to match patients who underwent PCI to patients who underwent CABG. Standardized mean difference was used to evaluate the balance before and after matching. A standardized difference of 0.1 or less was deemed to be the ideal balance and 0.1–0.2 was considered acceptable balance.

Forward selection with Cox proportional hazards regression models and Fine & Gray models were used for covariate selection. The entry threshold was set at *P* < 0.05. Candidate covariates were factors listed in Supplementary Table 1.

A sensitivity analysis of the time-dependent Cox model was conducted to examine the impact of immortal time bias due to the waiting period from the date of angiogram to the date of revascularization. All statistical analyses were executed using SAS 9.4 (SAS Institute, Cary NC).

### Reporting summary

Further information on research design is available in the Nature Portfolio Reporting Summary linked to this article.

## Results

**Fig. 1:**
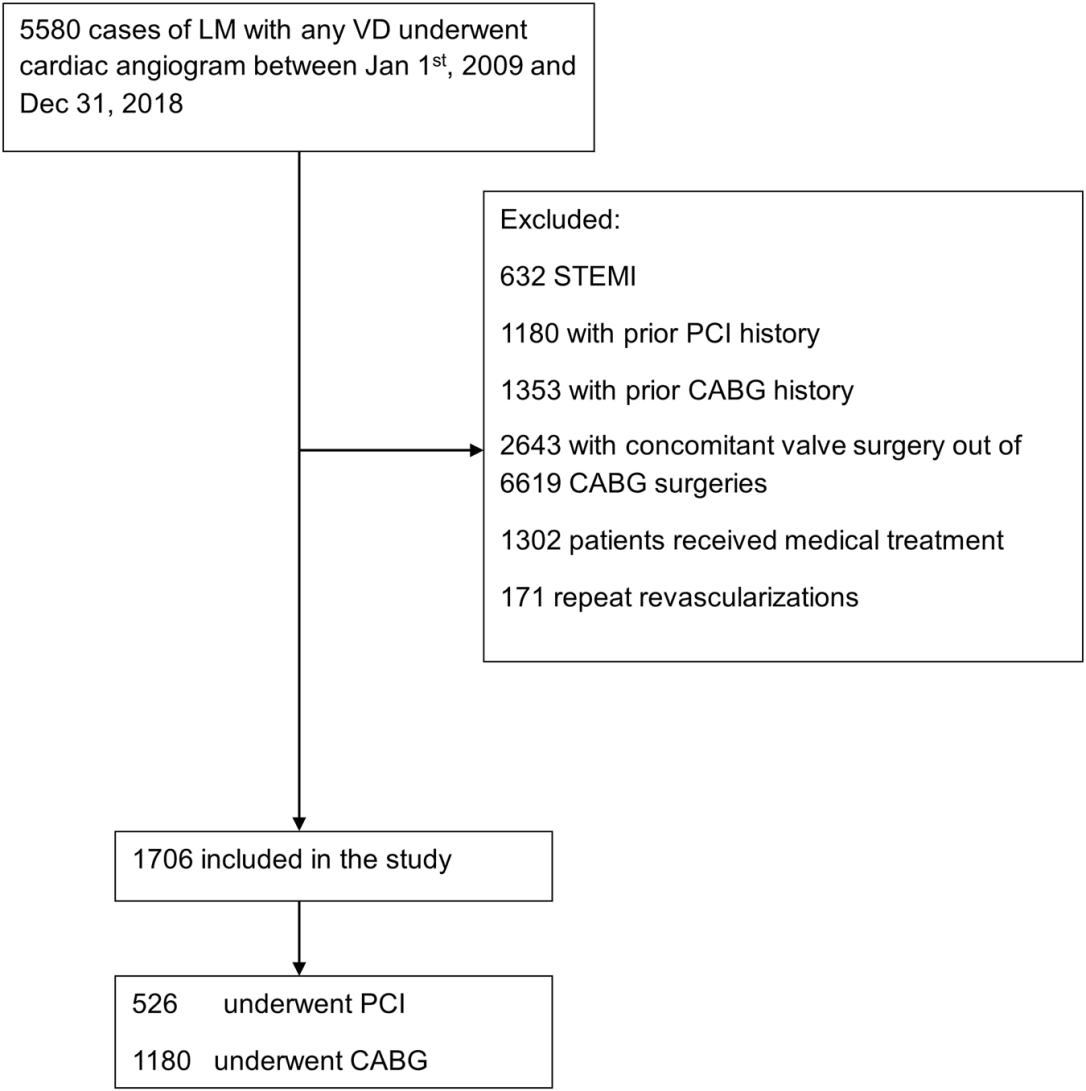
Flow diagram of patient inclusion and exclusion for the study. Flow diagram illustrating the inclusion of patients in this study including total number of patients initially identified, number of patients excluded with reasons for exclusion, and final number of patients in each group. LM left main, VD vessel disease, STEMI ST elevation myocardial infarction, PCI percutaneous coronary intervention, CABG coronary artery bypass grafting.

5580 patients were diagnosed with significant LMCAD on coronary angiography between January 1, 2009 and December 31, 2018. Patients were excluded for STEMI (623), prior PCI (1180), prior CABG (1353), concomitant valve surgery with CABG (2643), received medical therapy only (1302), and repeat revascularizations (171). 1706 patients were included in this study, 526 of whom underwent PCI (31%) and 1180 underwent CABG (69%) (Fig. 1).

### Baseline characteristics

Baseline characteristics of patients with LMCAD and any vessel disease (VD) that underwent CABG or PCI are shown in Table 1. CABG patients were significantly younger (67 ± 9 vs 71 ± 11, *p* < 0.001), with fewer females (15.3 vs 28.5, *p* < 0.001), fewer had heart failure (3.1% vs 8.4% *p* < 0.001), CKD (0.5% vs 2.1% *p* < 0.002), or malignancy (0.3% vs 2.3% *p* < 0.001). They were on fewer non-ASA antiplatelets (25.8% vs 35.6%, *p* < 0.001) and anticoagulants (6.9% vs 12.7% *p* < 0.001).

### Morbidity and mortality

**Fig. 2:**
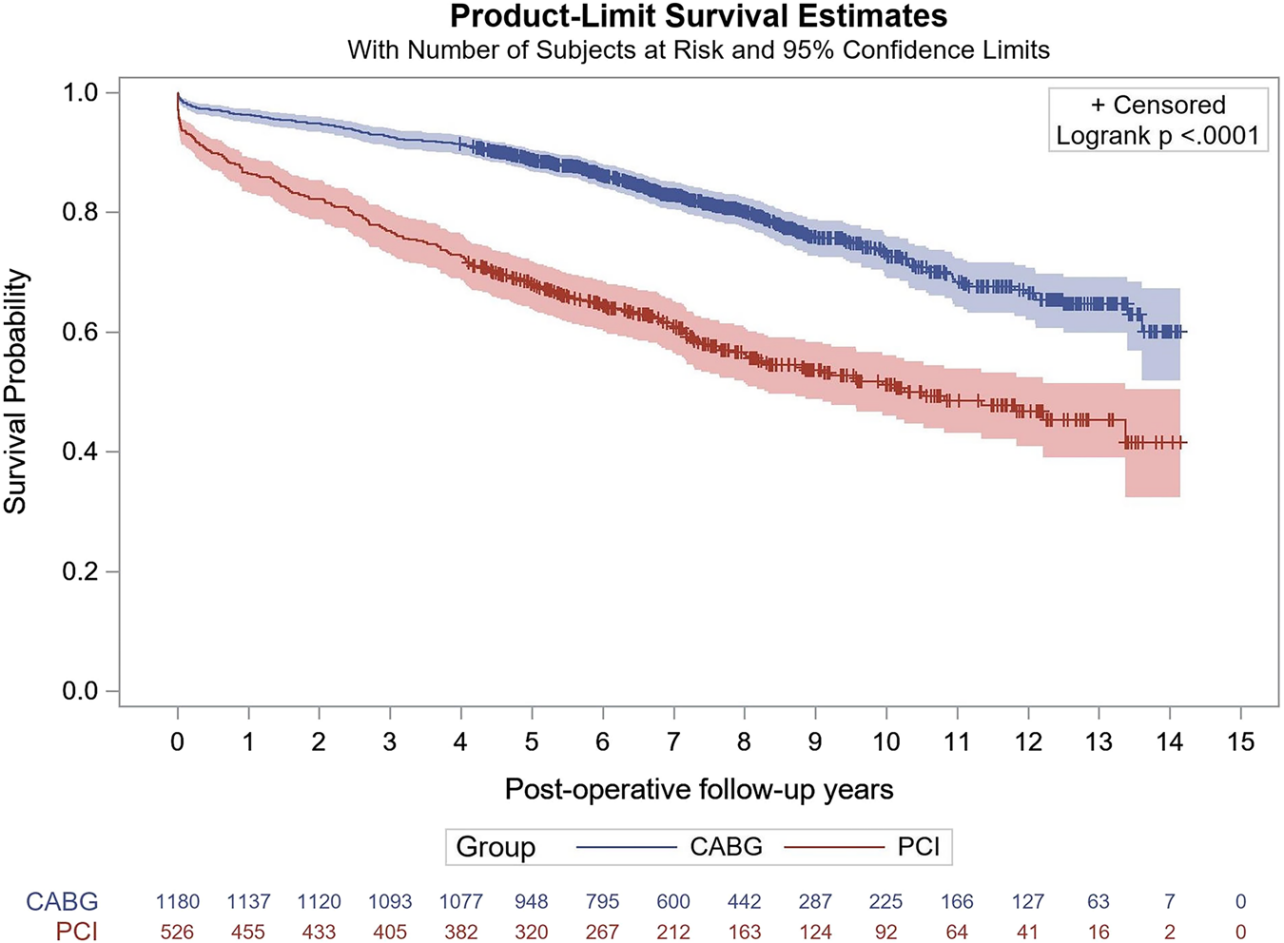
Kaplan–Meier curve for mortality among the study population. Kaplan–Meier Curve for death demonstrating higher rates of survival among patients who underwent CABG, included as the blue line, compared to patients who underwent PCI, included as the red line. CABG coronary artery bypass grafting, PCI percutaneous coronary intervention.

**Fig. 3:**
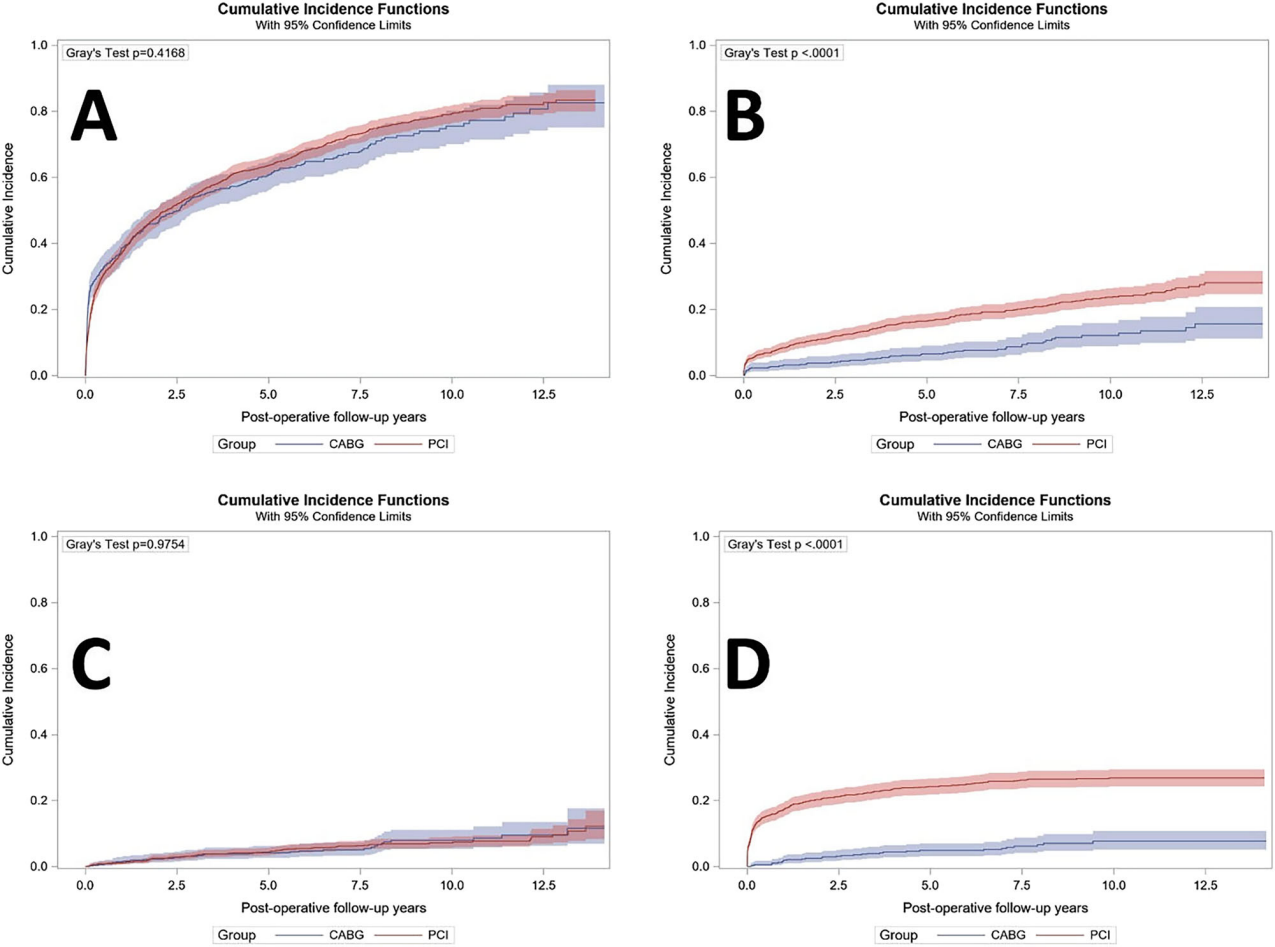
Cumulative incidence curve for non-fatal outcomes. Cumulative Incidence Curve for non-fatal outcomes up to 14.2 years with the blue line indicating CABG patients and the red line indicating PCI patients. **A** Readmission demonstrating no significant differences between groups; **B** Readmission for Myocardial Infarction which was significantly lower for the CABG patients compared to the PCI patients. **C** Readmission for Stroke demonstrating no significant differences between groups; **D** Repeat revascularization which was significantly lower for the CABG patients compared to the PCI patients. CABG coronary artery bypass grafting, PCI percutaneous coronary intervention.

All-cause mortality at the longest follow-up period of 14.1 years and mean follow-up of 7 years was significantly lower in patients undergoing CABG (40%) compared to PCI (58.4%) (*p* < 0.001)(Table 2, Fig. 2)(adjusted hazard ratio (aHR) 0.58, 95% confidence interval (CI) 0.48–0.70, *p* < 0.001). Rates of readmission for MI at the longest follow-up were also significantly lower in patients undergoing CABG (10.7%) versus PCI (22.3%)(aHR 0.40, 95% CI 0.29–0.55, *p* < 0.001) as well as rates of repeat revascularization for patients undergoing CABG (5.4%) versus PCI (16.3%) (aHR 0.25, 95% CI 0.18–0.36, *p *< 0.001). Rates of rehospitalization (*p* = 0.65) and readmission for stroke (*p* = 0.18) did not differ significantly between groups over the follow-up period. Patients with more than 1 rehospitalization included 470 (40%) of CABG patients and 281 (53%) PCI patients. Kaplan–Meier estimates mirrored these results with lower rates of readmission for MI (*p* < 0.0001)(Fig. 3B) and repeat revascularization (*p* < 0.0001)(Fig. 3D) in patients who had undergone CABG. There was similarly no evidence of differences in all-cause rehospitalizations (*p* = 0.4168) (Fig. 3A) or readmissions for stroke (*p* = 0.9754) (Fig. 3C).

**Table 2:** Comparison of outcomes between CABG and PCI for patients with LM with any vessel disease

Outcomes	CABG^a^ (*N* = 1180)	PCI^a^ (*N* = 526)	Adjusted HR^b^ (95% CI) (PCI as the reference group)	P-value for aHR
All cause death at 30 days follow-up	19(1.6%)	33(6.3%)	0.32(0.18, 0.60)	**0.0003**
30 days Readmission for MI	14(1.2%)	29(5.5%)	0.28(0.13, 0.56)	**0.0004**
All-cause death at longest follow-up (mean follow-up = 7 years)	253(40%)	231(58.4%)	0.58(0.48, 0.70)	**3.56e-08**
Rehospitalization (mean follow-up = 6.5 years)	711(75.6%)	352(71.6%)	0.97(0.84, 1.11)	0.645
Readmission for MI (mean follow-up = 6.5 years)	82(10.7%)	97(22.3%)	0.40(0.29, 0.55)	**1.23e-08**
Readmission for stroke (mean follow-up = 6.5 years)	63(8.3%)	22(5.7%)	1.41(0.85, 2.33)	0.183
Repeat revascularization(mean follow up = 6.5 years)	56(5.4%)	84(16.3%)	0.25(0.18, 0.36)	**8.74e-15**

Bold values indicate statistical significance.

*CABG* coronary artery bypass grafting, *PCI* percutaneous coronary intervention, *MI* myocardial infarction.

^a^The failure rate in the parentheses were estimates from the Kaplan–Meier curve or cumulative incidence curve at the longest follow-up.

^b^Adjusted based on the covariates listed in Table 1.

### Propensity score matched cohort

Following propensity score matching, 487 pairs of patients were included for the PCI and CABG. Following matching, baseline characteristics were similar between groups with ideal matching of patients identified by a standardized mean difference of <0.1 for all variables (Supplementary Tables 1, 2). The outcomes identified in the propensity scored matched comparisons were similar to the comparison of the overall cohort with all-cause death at longest follow-up (56.4% PCI vs 53.5% CABG, aHR 0.62, 95% CI 0.50–0.76, *p* < 0.001), readmission for MI (22.5% PCI vs 8.8% CABG, aHR 0.35, 95% CI 0.24–0.52, *p* < 0.001), and repeat revascularization (16.3% PCI vs 5.1% CABG, aHR 0.29, 95% CI 0.18–0.45, *p* < 0.001) favoring CABG. Rehospitalization for all causes and readmission for stroke were not significantly different between groups (Supplementary Table 3, Supplemental Figs. 1, 2).

### Trends of CABG and PCI over the inclusion period

From 2009 to 2018 the proportion of eligible patients with LMCAD undergoing CABG or PCI changed significantly (Table 3). In 2009, 24.4% of patients were referred for CABG while 14.4% of patients were referred for PCI and 61.2% were treated with medical therapy only. In 2018, 49.1% of patients were referred for CABG while 25.4% of patients were referred for PCI and only 25.4% were treated with medical therapy only. Overall, in the span of 9 years, both CABG and PCI saw an increase in referrals while the patients who received medical treatment alone declined.

**Table 3:** Trends in CABG and PCI rates among patients with LMCAD with any Vessel disease from 2009 to 2018

	CABG (*N* = 1180)	PCI (*N* = 526)
2009	71(24.4%)	42(14.4%)
2010	89(29.4%)	37(12.2%)
2011	51(16.9%)	58(19.2%)
2012	61(22.6%)	28(10.4%)
2013	71(22%)	53(16.4%)
2014	163(51.4%)	51(16.1%)
2015	177(56.5%)	63(20.1%)
2016	209(60.8%)	61(17.7%)
2017	149(56.9%)	61(23.3%)
2018	139(49.1%)	72(25.4%)

*CABG* coronary artery bypass grafting, *PCI* percutaneous coronary intervention.

## Discussion

Several important findings were identified in this investigation. In patients undergoing revascularization for LMCAD, CABG was associated with better long-term outcomes at 14 years compared to PCI. Patients who underwent CABG were less likely to experience readmission for MI, less likely to require repeat revascularization, and more likely to survive compared to patients who underwent PCI (Graphical Abstract). Rates of stroke and overall hospital readmission did not differ significantly between the groups. Interestingly, the short-term outcomes of 30-day mortality and MI at 30 days also favored CABG. This highlights that despite being a more invasive procedure, CABG can provide superior short-term outcomes when it is utilized in the appropriate patient population. Importantly, the low rates of early MI with CABG must be considered in the context of previous literature which has identified elevated rates of mortality associated with early post-CABG myocardial ischemia, reinforcing the importance of early graft patency, as well as the avoidance and early identification of myocardial ischemia post-revascularization^14^.

Several key trials have attempted to investigate the differences in outcomes with the available revascularization modalities for patients with LMCAD. While outcomes have generally favored CABG, the results have been inconsistent when considering certain outcomes or patient populations.

In 2019, the EXCEL Trial was published which randomized 1905 patients with LMCAD and low to either PCI or CABG. The trial followed patients for 5 years and found no significant differences in rates of the primary composite outcome of death, stroke, or myocardial infarction. The trial did find that patients who underwent PCI did have higher rates of death, ischemia-driven revascularization, and the composite of death, stroke, myocardial infarction, or ischemia-driven revascularization^9^. Several controversial points were felt to impact the results of the EXCEL Trial and explain the differences in outcomes with the NOBLE Trial. The EXCEL Trial utilized the Society for Cardiovascular Angiography and Interventions (SCAI) definition of peri-procedural MI rather than the Universal Definition of MI, which was the pre-specified definition and was subsequently changed to SCAI, which resulted in 37% higher incidence of MI in the CABG group. At face value, the significant differences in important outcome such as primary composite outcomes and MI results in heterogeneity between landmark studies on this topic, hindering the direct comparison between trials and aggregation of the data, further contributing to the discrepancies in the literature. The CABG group was also heterogenous, including on and off-pump CABG and various graft combinations in contrast to the PCI group which received the same treatment. Finally, despite the significantly higher incidence of revascularization, a secondary combined end-point of death, stroke, myocardial infarction, or ischemia-driven revascularization, and even mortality alone, PCI was still described as non-inferior to CABG^15–17^.

The NOBLE trial randomly assigned 1201 patients with LMCAD to either PCI or CABG and patients were followed for 5 years. Patients who underwent PCI experienced significantly higher rates of MI, repeat revascularization, and the composite primary outcomes of all-cause mortality, non-procedural MI, repeat revascularization, and stroke^10^. In the SYNTAX Trial published in 2009, 1800 patients with three-vessel CAD or LMCAD were randomly assigned to PCI or CABG. At 1 year, the composite outcome was significantly higher in the PCI group compared to CABG. Additionally, rates of repeat revascularization favored CABG while rates of stroke favored PCI. SYNTAX score was not associated with outcomes in the CABG group, but a high SYNTAX score was associated with increased incidence of the composite outcome for PCI^3^. In summary, despite the similarities between these three trials being improved rates of mortality and repeat revascularization for CABG, the discrepancies in primary composite outcomes and rates of MI, along with limited follow-up, have continued to drive controversies in this field.

Several meta-analyses have also attempted to address this point by pooling data and addressing the limitations of sample size. Takagi and colleagues pooled data from randomized control trials and propensity-matched observational studies for a total of 12,387 patients who underwent PCI or CABG for LMCAD. The pooled analysis found rates of the composite outcomes of death, myocardial infarction, and repeat revascularization favoring CABG, in the randomized control trials, observational studies, and overall pooled data^18^. A more recent meta-analysis by Sabatine et al. pooled data from randomized control trials with at least 5 years of follow-up comparing PCI and CABG for LMCAD. This meta-analysis included a total of 4 randomized control trials. While the rates of death did not differ significantly, rates of myocardial infarction and repeat revascularization favored CABG^19^.

The approaches to revascularization in previous studies and in the contemporary era are important to consider. Of the aforementioned trials, the SYNTAX was published the earliest. In the SYNTAX Trial, only first-generation drug-eluting stents were utilized^3^. In contrast, the NOBLE Trial utilized second-generation drug-eluting stents for the majority of patients undergoing PCI and the EXCEL Trial used second-generation stents for all patients undergoing PCI^9,10^. These differences are important to consider as second-generation drug-eluting stents are associated with improved outcomes and are more commonly used in the contemporary era. While stent generation was largely standardized within studies, CABG approach was not. In the aforementioned trials, coronary revascularization was performed based on local standard practice and allowed for arterial or venous grafts for revascularization and with or without cardiopulmonary bypass. In the SYNTAX and EXCEL trials, there was a strong recommendation for multiple arterial grafts for patients <70 years old although this was not enforced and was ultimately left to the surgeon’s discretion. In the SYNTAX Trial, 15% of patients underwent off-pump CABG and 19% received complete revascularization. In the EXCEL Trial, 30% of patients underwent off-pump CABG and 25% received only arterial conduits. While similar, these rates do differ between trials and the heterogeneity in revascularization approaches further limits the ability to directly compare the results. Future studies should aim to standardize revascularization techniques, aiming to utilize contemporary approaches to stent selection for PCI and conduit selection along with surgical technique in CABG, resulting in the best comparison between revascularization modalities.

While evidence supporting CABG in LMCAD has not been consistent across individual outcomes and separate studies, several trends have been demonstrated in the literature. In both randomized control trials and observational studies, rates of MI and repeat revascularization following treatment for LMCAD commonly favor CABG compared to both PCI and medical therapy, especially over the mid to long term^9,10,20,21^. Rates of mid to long-term mortality have either favored CABG or have been not significantly different. Despite strong evidence supporting CABG over PCI, PCI continues to be supported as a suitable alternative to CABG^22^. For example, the EXCEL Trial is often cited as presenting non-inferior outcomes for PCI compared to CABG given that non-inferiority was identified for the primary outcome, even with the previously mentioned limitations. The secondary outcomes are often disregarded as differences in rates of death, ischemia-driven revascularization, and secondary composite were significantly in favor of CABG^9,22–24^. Despite the evidence in support of CABG, whether for composite or individual outcomes, PCI is still commonly used in patients with LMCAD even in contemporary cohorts, as highlighted by this study. While CABG confers long-term benefits over PCI in patients with LMCAD, patients with increased surgical risk or limited life expectancy such as those with advanced age, significant comorbidities, or frailty may be at an elevated surgical risk or may not have a life expectancy that would allow for patients to benefit from the long-term superiority of CABG. These factors should be considered in the evaluation of patients with LMCAD as not all patients with this disease may be appropriate for surgical revascularization.

In addition to the described limitations of the literature, previous studies have also been limited due to either sample size or duration of follow-up. This investigation provides long-term follow-up with 14-year data from a real-world cohort and adjusted statistics demonstrating reduced rates of mortality, myocardial infarction, and required repeat revascularization with CABG compared to PCI. An additional strength of this study is the collection of data from a provincial database reducing the risk of readmissions or outcomes not being collected from peripheral sites, as is a potential risk in other locations where adverse outcomes may not be recorded if patients present to a hospital outside of their health maintenance organization. With these findings in mind, selection of revascularization strategies must be individualized to the patient taking into consideration their anatomy, comorbidities, and patient preference. A multidisciplinary Heart Team review is widely advocated for and ensures that the optimal intervention is pursued for each patient with LMCAD.

The retrospective nature of this study limits the ability to randomize patients to treatment and we cannot exclude residual confounding, although this has been addressed through statistical adjustments for each comparison. Over time practices have changed, including conduit choice for CABG with arterial grafts being more frequently utilized improving long-term outcomes, more recent generations of stents in PCI improving durability, and patient selection for both, which may influence outcomes by addressing shortcomings of approaches used in previous years. We are unable to determine the reason for referral to either treatment modality for individual patients (clinician choice, patient choice, resource limitations, etc) which can be addressed in future studies by specifically documenting the factors that resulted in the selected referral. Additionally, specific diagnoses of type 4 and 5 MI were not captured in the database, and comparisons of early graft failure between groups was not possible. We were unable to assess extent of concomitant disease as patients coded with LMCAD did not have the number of other diseased vessels recorded in the database and future studies should aim to collect and report detailed anatomic data which will help to differentiate patients treated with either revascularization modality allowing for more granular analyses. A clinical definition of heart failure was used in this study as left ventricular ejection fraction is inconsistently collected in the database used for this study. Finally, the data collected as part of this study was limited to a single center which may limit generalizability of the data due to specific approaches to patient referral, management, and follow-up in our region that may not be replicated in other healthcare settings.

While many previous studies have found benefits for LMCAD patients following CABG, this has not been consistent in every investigation. The majority of previous investigations into this topic have been limited by sample size or duration of follow-up. In this study, we provide long-term follow-up up to 14 years, among the longest of any large investigation into this topic, of a large cohort of patients undergoing PCI or CABG and have found significant benefits with CABG over PCI in terms of mortality, myocardial infarction, and required repeat revascularization. Future studies into this topic should aim to standardize revascularization techniques as the field develops, including the use of contemporary stents for PCI and conduit selection for CABG, resulting in the best comparison between revascularization modalities. While PCI is an indispensable tool in the management of CAD for patients with high surgical risk, low anatomic complexity, and significant comorbidities, and LMCAD treatment should always be individualized to recognize the specifics of each patient’s situation and coronary anatomy, CABG remains the gold standard revascularization therapy for patients with LMCAD and acceptable surgical risk for reducing the risk of mortality, MI, and repeat revascularization based on contemporary data and current guideline recommendations.

## Supplementary information

Supplemental Information

Reporting Summary

## Data Availability

The raw data associated with the article is not permitted to be shared externally as the data used in this study comes from a clinical database from which we have not received approval to share the data publicly. The data in this study originated from the APPROACH (Alberta Provincial Project for Outcome Assessment in Coronary Heart Disease) database and the Alberta Provincial Death Registry. Any requests to obtain access to the data should be directed to the Health System Access at research.administration@ahs.ca.
