# Supplementary material for: Revascularization strategy for left main coronary artery disease comparing percutaneous coronary intervention versus coronary artery bypass grafting
Source: Commun Med (Lond). 2025 Sep 29;5:402. doi: 10.1038/s43856-025-01098-w (PMC12480625; doi:10.1038/s43856-025-01098-w)
Supplement: Supplementary file 1 — Supplemental Information [file 43856_2025_1098_MOESM1_ESM.pdf]

Supplementary Table 1. Baseline characteristics of patients with LMCAD that underwent CABG or PCI before propensity score matching.

**Variables**	**CABG (N=1180)**	**PCI (N=526)**	**SMD**
Age, years	67 ± 9	71 ± 11	0.45
Female	181 (15.3)	150 (28.5)	0.32
Hypertension	690 (58.5)	306 (58.2)	0.01
Dyslipidemia	720 (61)	314 (59.7)	0.03
Heart failure	36 (3.1)	44 (8.4)	0.23
Atrial fibrillation	3 (0.3)	5 (1)	0.09
Type I or II Diabetes	301 (25.5)	143 (27.2)	0.04
COPD	67 (5.7)	43 (8.2)	0.1
CEVD	29 (2.5)	22 (4.2)	0.1
CKD	6 (0.5)	11 (2.1)	0.14
PAD	38 (3.2)	24 (4.6)	0.07
Any Liver disease	0 (0)	1 (0.2)	0.06
Malignancy	4 (0.3)	12 (2.3)	0.17
Current smoker	228 (19.3)	78 (14.8)	0.12
Former smoker	326 (27.6)	145 (27.6)	<0.01
**Baseline Medications**			
ASA	787 (66.7)	348 (66.2)	0.01
Other antiplatelets	305 (25.8)	187 (35.6)	0.21
Statin	626 (53.1)	290 (55.1)	0.04
ACEi/ARB	569 (48.2)	277 (52.7)	0.09
Beta-blocker	634 (53.7)	300 (57)	0.07
Anticoagulant	81 (6.9)	67 (12.7)	0.2

Supplementary Table 2. Baseline characteristics of patients with LMCAD that underwent CABG or PCI after propensity score matching.

**Variables**	**CABG (N=487)**	**PCI (N=487)**	**SMD**
Age, years	70 ± 9	71 ± 11	0.09
Female	128 (26.3)	122 (25.1)	0.03
Hypertension	271 (55.6)	274 (56.3)	0.01
Dyslipidemia	277 (56.9)	281 (57.7)	0.02
Heart failure	27 (5.5)	30 (6.2)	0.03
Atrial fibrillation	3 (0.6)	3 (0.6)	<0.01
Type I or II Diabetes	121 (24.8)	124 (25.5)	0.01
COPD	33 (6.8)	31 (6.4)	0.02
CEVD	20 (4.1)	19 (3.9)	0.01
CKD	6 (1.2)	6 (1.2)	<0.01
PAD	21 (4.3)	23 (4.7)	0.02
Any Liver disease	0 (0)	0 (0)	<0.01
Malignancy	4 (0.8)	4 (0.8)	<0.01
Current smoker	81 (16.6)	76 (15.6)	0.03
Former smoker	128 (26.3)	134 (27.5)	0.03
**Baseline Medications**			
ASA	314 (64.5)	320 (65.7)	0.03
Other antiplatelets	161 (33.1)	159 (32.6)	0.01
Statin	257 (52.8)	261 (53.6)	0.02
ACEi/ARB	240 (49.3)	252 (51.7)	0.05
Beta-blocker	261 (53.6)	274 (56.3)	0.05
Anticoagulant	44 (9)	52 (10.7)	0.06

Supplementary Table 3. Comparison of long-term outcomes between CABG and PCI for patients with LM with any vessel disease.

Outcomes	CABG* (N=487)	PCI* (N=487)	HR(95% CI) (PCI as the reference group)	P value for HR
All-cause death at longest follow-up	141(53.5%)	203(56.4%)	0.62(0.50, 0.76)	**1.08e-05**
Rehospitalization	334(80.6%)	321(70.9%)	1.03(0.88, 1.20)	0.714
Readmission for MI	33(8.8%)	89(22.5%)	0.35(0.24, 0.52)	**2.14e-05**
Readmission for stroke	28(6.8%)	20(5.7%)	1.43(0.81, 2.54)	0.217
Repeat revascularization	24(5.1%)	78(16.3%)	0.29(0.18, 0.45)	**7.92e-08**

CABG: coronary artery bypass grafting. PCI: percutaneous coronary intervention. MI: myocardial infarction.

*The failure rate in the parentheses were estimates from the Kaplan Meier curve or cumulative incidence curve at the longest follow-up.

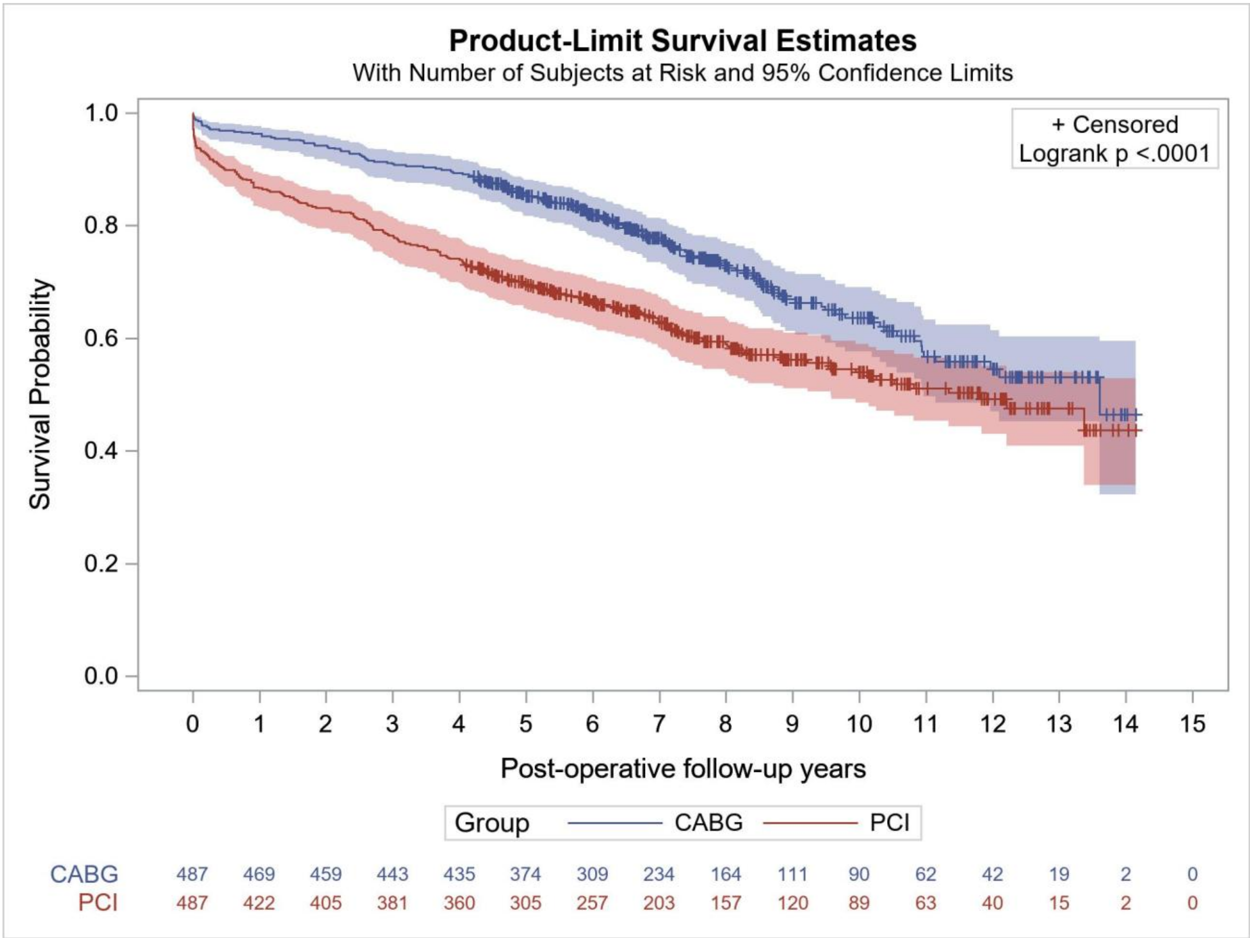

Supplementary Figure 1: Kaplan-Meier Curve for all-cause death at the longest follow up after propensity score matching

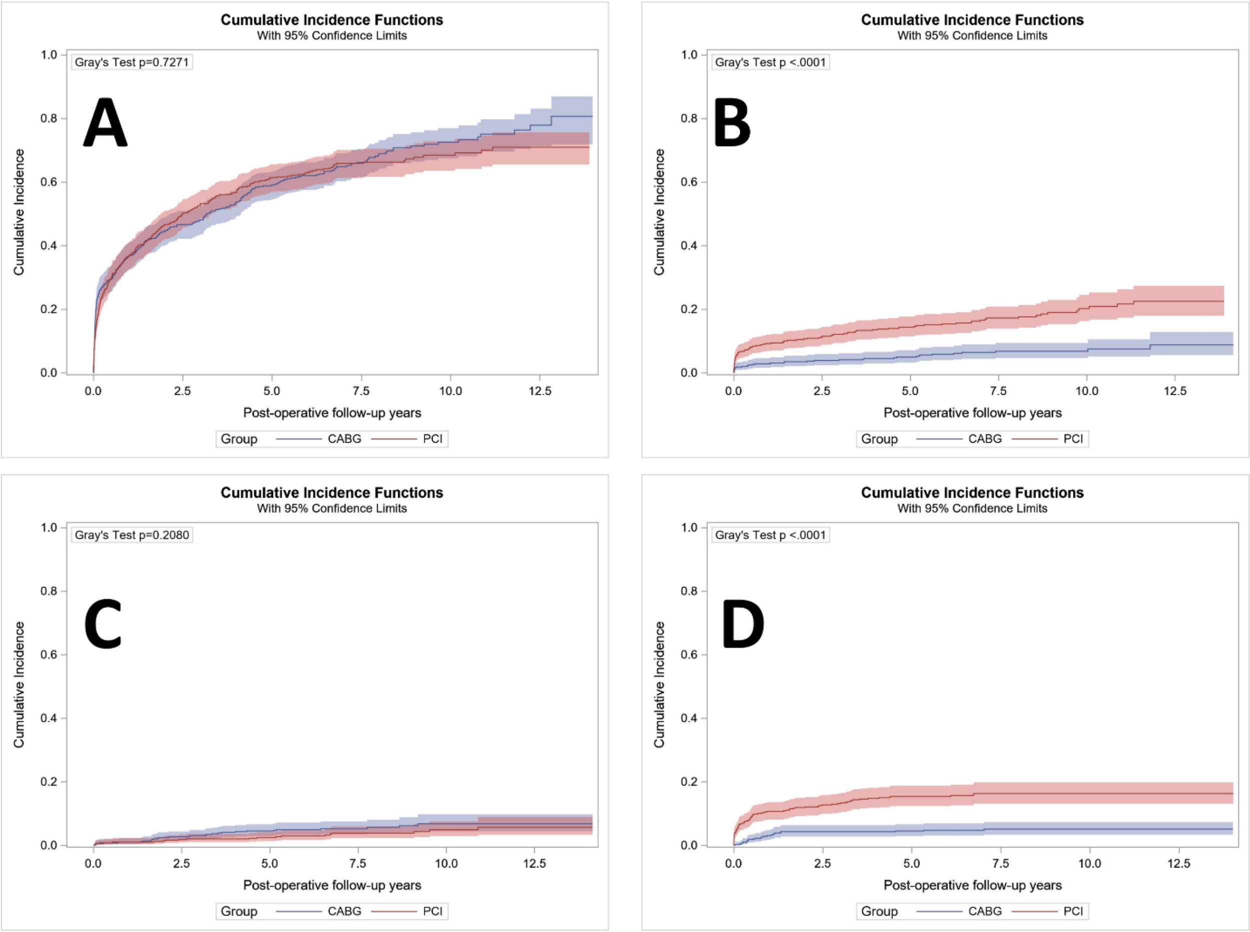

Supplementary Figure 2. Cumulative Incidence Curve for non-fatal outcomes for the longest follow up after propensity score matching

A: Readmission; B: Readmission for Myocardial Infarction C: Readmission for Stroke; D: Repeat revascularization
